# Dietary Supplementation with Nucleotides, Short-Chain Fructooligosaccharides, Xylooligosaccharides, Beta-Carotene and Vitamin E Influences Immune Function in Kittens

**DOI:** 10.3390/ani13233734

**Published:** 2023-12-02

**Authors:** Jujhar Atwal, Willy Joly, Robyn Bednall, Fabio Albanese, Michelle Farquhar, Lucy J. Holcombe, Phillip Watson, Matthew Harrison

**Affiliations:** 1 WALTHAM Petcare Science Institute, Melton Mowbray, Leicestershire LE14 4RT, UK; robyn.bednall@effem.com (R.B.); fabio.albanese@effem.com (F.A.); michelle.farquhar@effem.com (M.F.); lucy.holcombe@effem.com (L.J.H.); phillip.watson@effem.com (P.W.); matt.x.harrison@effem.com (M.H.); 2 Royal Canin SAS, 650 Avenue de la Petite Camargue, 30470 Aimargues, France

**Keywords:** nucleotides, short-chain fructooligosaccharides, xylooligosaccharides, immune, kitten, feline, vaccination, humoral

## Abstract

**Simple Summary:**

Newborns are susceptible to infectious disease, and early life represents a critical developmental window of the immune system, which is important for preventing and fighting disease. There is evidence that nucleotides, oligosaccharides and vitamins can positively influence immune function; however, the evidence that is specifically applicable to cats is limited. This current study fed domestic short hair kittens either a dry format control diet or a test diet fortified with nucleotides, short-chain fructooligosaccharides, xylooligosaccharides, β-carotene and vitamin E until the kittens reached 28 weeks of age. The kittens followed a routine preventative medication schedule for vaccination and a suite of health, metabolism and immune parameters were measured at regular intervals throughout the study. All kittens remained healthy and did not display any clinically relevant signs of adverse health. Antibodies are produced following infection or vaccination. The level of antibody, the proportion of kittens who demonstrated an acceptable response to vaccination and the proportion of kittens who reached the recognised protective level of antibody were greater in kittens fed the test diet. The test diet group demonstrated a stronger antibody-mediated response to vaccination. Antibodies play a role in preventing disease; thus, this suggests that the test diet supports immune defence against immune challenges.

**Abstract:**

Nucleotides, short-chain fructooligosaccharides (scFOS), xylooligosaccharides (XOS), β-carotene and vitamin E are reported to enhance immune function; however, the evidence of this in cats is limited. The aim of this study was to determine the immunomodulatory effects of these ingredients in kittens. Forty domestic short hair kittens were designated in litters to control or test diet for 28 weeks. Test diet was fortified with 0.33 g nucleotides, 0.45 g scFOS, 0.3 g XOS, 0.7 mg β-carotene and 66.5 mg vitamin E per 100 g diet. Kittens were vaccinated against feline parvovirus (FPV) and herpesvirus (FHV) at 10, 14 and 18 weeks. Kittens remained healthy, with no measured evidence of adverse health. Serum FPV and FHV antibody titres were significantly (*p* < 0.05) higher in the test diet group at week 23 and 27, respectively. A significantly (*p* < 0.05) higher proportion of test diet group kittens demonstrated an adequate response (four-fold titre increase) to FHV vaccination and a significantly (*p* < 0.05) higher proportion reached a protective antibody titre for FHV. Serum IgM was significantly (*p* < 0.05) higher in the test diet group. The test diet group demonstrated a stronger humoral immune response to vaccination, suggesting the diet supports immune defence, enabling a greater response to immune challenges.

## 1. Introduction

The immune system is a complex layered system involved in both defence and healing, and its role in preventing and fighting diseases establishes it as a good indicator of overall health. The immune system is immature during the first year of life, and hence, offsprings are susceptible to infectious disease. Mammals have evolved to compensate this with passive transfer, such as the transfer of maternally derived antibodies (MDA) via both the placenta and colostrum/milk during lactation; however, this protection wanes over time [1]. The immune system develops during early life and can be influenced by nutrition. Nutrients can exert positive effects [1,2] both directly, via nutrient uptake and sensing via immune cells [3,4], and indirectly, via the reciprocal interactions of the intestinal microbiota and immune system [5]. There is evidence that nucleotides, oligosaccharides and vitamins can positively influence immune health.

Nucleotides are essential for cellular metabolism and participate in various biological processes including DNA and RNA synthesis, signalling, energy transfer, acting as co-enzymes, and have been shown to modulate immune function [6,7]. Nucleotides are found in maternal milk [6,8]; therefore, they likely play a role in offspring development. There is considerable evidence from human and animal studies that shows nucleotide supplementation has a favourable effect on immune indices such as response to immune challenges [6,8,9,10], increased immunoglobulin (IG) concentrations [8,11,12] and lymphocyte proliferation [8,10,13,14]. Clinical studies of ill patients fed a cocktail of ingredients, including nucleotides, found a reduction in hospitalisation time and reduction in infection and wound complications [14]. Other studies have shown that dietary nucleotides can reduce the risk of infection [15] and reverse immunosuppression [16].

Fructooligosaccharides (FOS) are non-digestible oligosaccharides composed of mainly fructose units linked by β2-1 bonds and are considered as prebiotics, having an ability to selectively stimulate the growth of health-associated bacteria in the colon such as bifidobacterium [17]. Bifidobacteria exert positive effects on various aspects of immune function, including the promotion of macrophages and the stimulation of antibody production [18]. Short-chain FOS (scFOS) is a subclass of FOS that have a smaller degree of polymerization (3–5). Whilst the evidence for scFOS supporting immune health is limited in companion animals, feeding scFOS to beagle dogs during gestation resulted in higher maternal milk IG levels [19].

Xylooligosaccharides (XOS) are also naturally derived prebiotics; these are non-digestible sugar oligomers and consist of a xylose backbone, linked by β1-4 bonds [20]. XOS can be fermented to short-chain fatty acids in the lower gastrointestinal tract [21], which promotes the growth of health-associated bifidobacterium and lactobacilli [22,23]. In chickens, XOS reduced salmonella colonisation in a dose-dependant manner [24], and *in vitro* studies have confirmed that XOS decreases the adherence of this pathogen to intestinal epithelial cells [25]. Whilst evidence linking XOS to immune modulation is limited, studies have demonstrated that XOS enhances the proliferative response of lymphocytes [26] and downregulates the inflammatory response [27].

Vitamin E is an essential nutrient and antioxidant, which increases cell proliferation [28] and enhances humoral immune responses [29] in animals. Dietary β-carotene is taken up by immune cells [30] and can positively influence cell-mediated and humoral immune responses [30,31] in companion animals.

As early life is a critical developmental window of the immune system, which can be influenced by nutrition, the purpose of the current study was to determine if a cocktail of functional ingredients that includes nucleotides, scFOS, XOS, β-carotene and vitamin E could modulate immune function in domestic short hair kittens over a period of 28 weeks.

## 2. Materials and Methods

### 2.1. Study Design

In a parallel study design, forty domestic short hair kittens were designated in litters to one of the two diet groups, with twenty kittens each (control or test). Kittens were offered one of the two dry format diets, differing in target nucleotides, scFOS, XOS, β-carotene and vitamin E levels (control and test) from the first feeding of solid diet (3 weeks of age) until 28 weeks of age. Pet caretakers and individuals completing sample or data analysis were blinded to the treatment group. The research protocol was reviewed and approved by the Waltham Animal Welfare and Ethical Review Body (on 12 March 2018) and carried out under the authority of the Animals (Scientific Procedures) Act (1986).

### 2.2. Animals and Housing

Domestic short hair kittens were bred and housed at the Waltham Petcare Science Institute, and only kittens considered healthy were included in the study. Kitten enrolment was staggered over 14 months, and whole litters were randomised at the litter level to either control or test diet group at birth due to housing and feeding logistics. The control diet group had eight females and twelve males from a total of seven litters, and the test diet group also had eight females and twelve males from a total of six litters. Kittens were housed with mothers in litters for up to 12 weeks in accommodation which provided a minimum of 3.4 m^2^ space and 2 m height. From 12 weeks, kittens were housed in social rooms which provided a minimum of 1.5 m^2^ floor and 0.5 m^2^ shelf area for the first kitten plus 0.75 m^2^ floor and 0.5 m^2^ shelf area per additional kitten per room. All rooms provided a minimum of 2 m height. Kittens were separated by sex by 18 weeks. Social rooms contained shelving at different heights and movable furniture, varied enrichment was provided daily such as different toys and exposure to different scents and sounds. All kittens had daily group socialisation with other cats and animal carers and training sessions for sample collection protocols.

From 3 weeks onwards, kittens had access to dry format diet *ad libitum* (control or test) in a group setting and weaning was completed for all kittens between 8 and 12 weeks. From 12 weeks, kittens had three individual meals per day including an overnight feed. To enable individual feeding, kittens received daytime meals for 20 min within individual feeding boxes (measuring 51 cm × 55 cm × 60 cm (height × length × depth)) and in individual microchip feeders (Surefeed; Sure Petcare, Clearwater, FL, USA) for overnight meals. Any daytime diet refusal was reoffered with the overnight feed, and any diet refused overnight was recorded as refusal. Overnight feeding was gradually withdrawn from 16 weeks, and all three meals were offered during the day. Diet was offered in amounts to maintain an ideal bodyweight (BW) and body condition score (BCS) according to a 9-point scale [32], and this was reviewed and adjusted (if required) on a weekly basis. Tap water was always freely available.

### 2.3. Vaccination

Core kitten vaccination followed a routine preventative medication schedule, and kittens were vaccinated with subcutaneous injection of Fevaxyn Pentofel (Zoetis UK Ltd., Leatherhead, UK) at 10, 14 and 18 weeks (±1 week). This adjuvanted vaccine contained inactivated feline Panleukopenia, i.e., Parvovirus (strain CU4), inactivated feline Leukaemia virus (strain 61E), inactivated feline Rhinotracheitis virus; Herpesvirus (strain 605), inactivated feline Calicivirus (strain 255) and inactivated feline Chlamydophila felis (strain Cello). 

### 2.4. Diets

Extruded dry format experimental diets were specifically formulated and manufactured for this study (Royal Canin, Aimargues, France). Two batches of control diet were used consecutively due to diet shelf life, whilst a single batch of test diet was used during this study. The second batch of control diet was fed to the last 7 control diet group kittens recruited for the study, during their last 20–60 days in the study. Control and test diets used the same core recipe consisting of poultry protein (dried), animal fat, maize flour, rice, vegetable protein isolate, animal protein (hydrolysed), fish oil, soya oil, beet pulp and vegetable fibres. The test diet additionally contained 0.33 g yeast extract rich in nucleotides (PetMOD, Prosol, Madone, Italy), 0.45 g scFOS (Profeed, Tereos, Moussy-le-Vieux, France), 0.3 g XOS (XOS 35, Longlive, Qingdao, China) and 0.7 mg β-carotene per 100 g of diet. The test diet contained 109.6 mg vitamin E per 4184 kJ (1000 kcal), whereas the control diets contained 49.8 and 57.5 mg vitamin E per 4184 kJ (1000 kcal) for the first and second batch, respectively. Nutrient composition analysis of the diets was completed at Eurofins Ltd. (Wolverhampton, UK) post-production (Table 1).

With the exception of choline, diets were compliant with Association of American Feed Control Officials (AAFCO) [33] and National Research Council (NRC) [34]. The requirement for choline can be substituted by the presence of other methyl donors such as methionine when in excess of the minimum requirement [35]. The combination of choline and excess methionine levels provided the NRC recommended allowance [34] for choline for the first batch of control and test diets, and therefore, supplementation was not needed. Daily choline chloride supplementation (Metabolics Ltd., Devizes, UK) was added to the second batch of control diet to provide a total of 637 mg per 4184 kJ (1000 kcal).

Commercial diets formulated for young kittens and queen cats (Royal Canin, Aimargues, France) were mainly used for feeding the queens during late gestation and lactation in this study.

### 2.5. Measures

#### 2.5.1. Intake, Bodyweight and Body Condition Score

Diet intake was recorded daily per individual kitten as mass (g) of diet offered minus mass (g) of diet refused. BW was recorded weekly (kg) on a digital weighing platform (Mettler Toledo, Columbus, OH, USA, IC5429). BCS was recorded weekly by trained scorers using a 9-point scale [32].

#### 2.5.2. Blood-Based Measures

A morning fasted blood sample (at least 3.5 h since last meal) was drawn using a needle and syringe from the jugular vein for blood-based analysis within one week of kittens reaching 10, 12, 14, 18, 23 and 27 weeks of age. Furthermore, 3, 4, 3, 4.7, 3 and 4.7 mL of blood was collected at weeks 10, 12, 14, 18, 23 and 27, respectively. Samples were decanted into blood tubes prior to being processed and analysed as detailed below and in Supplementary Table S1. For serum-based measures, serum was separated from blood in gel-activated serum clot tubes by standing for 30 min at room temperature followed by centrifugation (1999× *g* for 10 min at room temperature). Serum was used immediately to measure health biochemical parameters; otherwise, it was aliquoted and stored at −80 °C prior to antibody titre, cytokine and IG analyses.

#### 2.5.3. Serum Antibodies

Antibody titre and IG analysis was completed at all timepoints. FPV titres were determined using the widely used haemagglutinin inhibition (HI) test [36] validated by IDEXX bioanalytics (Kornwestheim, Germany). Sera was initially diluted 1:10, serially diluted two-fold (11 dilutions in total; 1:10–1:10,240) and incubated with a constant concentration of FPV, followed by an addition of a 0.5% solution of porcine red blood cells (RBCs). The serum dilutions were examined for RBC hemagglutination. Serum FPV antibody titres were determined as the reciprocal of the highest dilution of serum able to inhibit RBC hemagglutination.

FHV titres were determined using the widely used viral neutralisation test [37,38] validated by IDEXX bioanalytics (Kornwestheim, Germany). Sera was initially diluted 1:8 and 1:12, and each dilution was serially diluted two-fold (11 dilutions in total; 7 serial dilutions 1:8–1:512 and 4 serial dilutions 1:12–1:96) and incubated with a constant concentration of FHV, followed by an addition of a Madin-Darby Canine Kidney (MDCK) cell suspension. After four days, serum FHV antibody titres were determined via light microscopy as the reciprocal of the highest dilution of serum able to inhibit the cytopathic effect of FHV in MDCK cells.

FCV titres were determined using a validated feline calicivirus enzyme-linked immunosorbent assay (ELISA) kit (European Veterinary Laboratory, Woerden, Netherlands, EIA F1008-AB0), and analysis was completed by VetAgro Sup; Laboratoire Leptospires et Analyses Veterinaires (Marcy L’Etoile, France). Serum samples and negative and positive controls were diluted 1:30 in buffer and then serially diluted three-fold (four dilutions in total; 1:30–1:810) in buffer. Samples and controls were loaded in microtiter strips coated with anti-FCV antibodies and incubated at 37 °C for 60 min. Strips were then washed prior to the addition of 100 µL Horseradish Peroxidase labelled detection antibody and incubated at 37 °C for 60 min. After the washing steps, 100 µL of substrate was added to all the wells and incubated in the dark at room temperature for 15 min. A 50 µL volume of stop solution was added, and absorbency values (OD) were determined (within 10 min) at 450 nm, using 620 nm as a reference. FCV titre was calculated by constructing a curve and using cut-off line (8 × three-fold serial dilutions in total; 1:30–1:65,610) of OD on *y*-axis and titre on *x*-axis. ELISA titres were calculated using as cut-off that is 2.5 times of the OD value of negative control at 1:30. 

IgG, IgA and IgM were analysed using validated ELISA kits (IgG—Abcam, Cambridge, UK, ab190523; IgA—Abcam, Cambridge, UK, ab190547; IgM—LifeDiagnostics, West Chester, PA, USA, IGM-8).

#### 2.5.4. Serum Cytokines

Serum collected at 12, 14 and 23 weeks were analysed for FMS-related tyrosine kinase 3 ligand (Flt-3L), interferon gamma (IFN-γ), granulocyte-macrophage colony-stimulating factor (GM-CSF), interleukin-1 beta (IL-1β), interleukin-2 (IL-2), platelet-derived growth factor-BB (PDGF-BB), interleukin-12 (IL-12), interleukin-13 (IL-13), interleukin-4 (IL-4), interleukin-6 (IL-6), interleukin-8 (IL-8), keratinocyte chemoattractant (KC), stromal cell-derived factor 1(SDF 1), regulated on activation normal T-cell expressed and secreted (RANTES), stem cell factor (SCF), tumour necrosis factor alpha (TNFα) and interleukin-18 (IL-18) using the Milliplex feline cytokine/chemokine kit (Merck, Burlington, MA, USA, FCYTMAG-20K-PMX) on the Luminex 200 (Luminex Corporation, Austin, TX, USA). 

#### 2.5.5. Serum Biochemistry

Serum collected at all timepoints were analysed for alanine aminotransaminase (ALT), albumin, alkaline phosphatase (ALP), aspartate aminotransferase (AST), calcium, chloride, cholesterol, creatinine, glucose, inorganic phosphorus, potassium, sodium, total protein, triglycerides and urea using a AU480 biochemistry analyser (Beckman Coulter, Brea, CA, USA). 

#### 2.5.6. Lymphocyte Proliferation

Lithium heparin (0.3 mL)-treated whole blood collected at 12, 18 and 27 weeks was diluted 1:10 with complete media without glutamine and phenol red (RPMI 1640—Life Technologies, Carlsbad, CA, USA, 32404-014), 10% foetal bovine serum (Life Technologies, Carlsbad, CA, USA, 10270-106), 2 mM/mL L-glutamine (Life Technologies, Carlsbad, CA, USA, 25030-024) and 100 U/mL penicillin/100 µg/mL streptomycin (Life Technologies, Carlsbad, CA, USA, 15140-122). A 100 µL volume of sample was added to a round bottom plate (Corning, Corning, NY, USA, 3799). Concanavalin A (Con A), Phorbol 12-myristate 13-acetate/Ionomycin (PMA/ION) and Staphylococcal Enterotoxin B (SEB) were added to samples and incubated for 93–96 h at 37 °C, 5% CO_2_. A total of 8.79 and 17.78 µg/mL Con A (Sigma, St. Louis, MO, USA, C0412-5MG), 0.0125/0.125 and 0.0375/0.375 µg/mL PMA/ION (Sigma, St. Louis, MO, USA, P1585-10MG; Sigma, St. Louis, Missouri, USA, I0634-5MG) and 0.001 and 0.01 µg/mL SEB (Sigma, St. Louis, MO, USA, S4881-5MG) were added for EC50 and EC80 concentrations, respectively. Furthermore, 50 µL of blood and mitogen sample were transferred to a deep well plate (Eppendorf, Hamburg, Germany, EP-951032603). A 100 µL volume of mouse anti-cat antibody CD5-PE (Biorad, Hercules, CA, USA, MCA2038PE) or mouse IgG1 isotype control for PE (Biorad, Hercules, CA, USA, MCA1209PE) diluted 1:20 in StemPro^®^ Accutase^®^ Cell Dissociation Reagent (Life Technologies, Carlsbad, CA, USA, A11105-01) was added and incubated for 30 min at room temperature. A total of 100 µL of lysis buffer (red cell lysis buffer (Miltenyi biotec, Bergisch Gladbach, Germany, 130-094-183) diluted 1:2 with distilled water (Life Technologies, Carlsbad, CA, USA, 15230162) plus 0.3 µL/mL of Hoescht (Life Technologies, Carlsbad, CA, USA, H3570)) was added and incubated for 10 min at room temperature on a plate shaker. Then, 250 µL of storage solution (Miltenyi biotec, Bergisch Gladbach, Germany, 130-092-748) was added, and the plates were analysed for Hoescht+CD5+ events on a MACSQuant^®^ 9 flow cytometer (Miltenyi Biotec, Bergisch Gladbach, Germany).

#### 2.5.7. Phagocytosis

Lithium heparin (0.9 mL)-treated whole blood collected at 12, 18 and 27 weeks was transferred in 100 µL aliquots to the bottom of polypropylene tubes (Scientific Laboratory Supplies, Hessle, UK, TUB0106). The pHrodo™ BioParticles™ phagocytosis kit was used (Life Technologies, Carlsbad, CA, USA, A10025), and 20 µL of pHrodo™ Red E. coli BioParticles^®^ was added to half of the aliquots. Samples were incubated at either 37 °C or 4 °C for 15 min before all samples were placed on ice. A 100 μL volume of lysis buffer was added to all tubes, and samples were vortexed and incubated for 5 min at room temperature. A 1 mL volume of buffer was added to all tubes, and samples were vortexed and incubated for 5 min at room temperature before centrifugation (350× *g* for 5 min at room temperature). Supernatant was removed, and the pellet resuspended in 1 mL of wash buffer. Supernatant was removed following a second centrifugation (350× *g* for 5 min at room temperature) before the pellet was resuspended in 0.5 mL of wash buffer. The samples were analysed for PE+ events on a MACSQuant^®^ 9 flow cytometer (Miltenyi Biotec, Bergisch Gladbach, Germany).

#### 2.5.8. Haematology

K3-EDTA (0.2 mL)-treated whole blood collected at 10, 14, 18, 23 and 27 weeks was assessed for complete blood count (leucocyte and erythrocyte counts, haemoglobin concentration, haematocrit, platelet count, mean corpuscular volume, mean corpuscular haemoglobin, lymphocyte count, monocyte count and granulocyte count) using a Mythic 18 haematology analyser (Orphée SA, Geneva, Switzerland).

#### 2.5.9. Nucleotides

K3-EDTA (0.6 mL)-treated whole blood collected at 10, 18 and 27 weeks was combined with 2.4 mL methanol (Sigma, St. Louis, MO, USA, 322415-1L) prior to storage at −80 °C. The samples were shipped on dry ice to NovoCIB (Boulogne-sur-Mer, France) to complete their extraction. Briefly, 30% ammonium hydroxide was added prior to a 24 h incubation and centrifugation (13,000× *g* for 10 min). Extracted nucleotides were quantified using ion-paired reverse-phase high-performance liquid chromatography, UV-HPLC [39]. A subset of samples was further analysed by dephosphorylation, converting nucleotides to nucleosides before analysis.

#### 2.5.10. Phenotyping

K3-EDTA (0.5 mL)-treated whole blood collected at 18 and 27 weeks was added to tubes (Scientific Laboratory Supplies, Hessle, UK, TUB0106) in 20 µL aliquots prior to the addition of 20 μL of antibodies (mouse anti-cat CD5 (Biorad, Hercules, CA, USA, MCA2038F or MCA2038PE), CD4 (Biorad, Hercules, CA, USA, MCA1346F), CD8 (Biorad, Hercules, CA, USA, MCA1347PE), mouse anti-dog CD21 (Biorad, Hercules, CA, USA, MCA1781A647) and CD18 (Biorad, Hercules, CA, USA, MCA1780A647)) diluted 1:10 in PBS without CaCl_2_ and MgCl_2_ (Sigma, St. Louis, MO, USA, D8537) and 0.1% BSA (Thermofisher, Waltham, MA, USA, 15260037). Unstained cells were included as a negative control, and single-stained cells were used to determine spectral overlap and apply appropriate compensation settings. Isotype controls were utilised to confirm the specificity of antibody staining and fluorescence minus one (FMO) control were included to reveal background fluorescence and identify positive populations for accurate gating. Tubes were vortexed and incubated for 30 min at room temperature before 197 μL of buffer (red cell lysis buffer (Miltenyi biotec, Bergisch Gladbach, Germany, 130-094-183) diluted 1:2 in distilled water (Life Technologies, Carlsbad, CA, USA, 15230162) plus 0.3 μL/mL Hoechst 33,342 (Life Technologies, Carlsbad, CA, USA, H3570)) was added. Tubes were vortexed and incubated for 10 min at room temperature before 750 μL of storage solution (Miltenyi biotec, Bergisch Gladbach, Germany, 130-092-748) was added, and samples were vortexed before analysis on the MACSQuant^®^ 9 flow cytometer (Miltenyi Biotec, Bergisch Gladbach, Germany).

#### 2.5.11. Physical Measurements

Physical measurements of each kitten’s height at withers, thoracic girth and rump width (cm) were taken using a tape measure at 28 weeks to assess growth. 

#### 2.5.12. Statistical Powering

Study powering was performed for the primary response variable, lymphocyte proliferation by simulation, using data generated from an *in vitro* study that screened the effect of oligosaccharides on lymphocyte proliferation (Unpublished work). Results indicated 20 kittens were required in each group, the control and test diet groups, for 80% power and for an effect size of 5–20% for the mitogens at EC50 and EC80. 

#### 2.5.13. Statistical Analysis

The data were analysed using linear mixed effects models with each measure as the response. Fixed effects of diet, age and their interaction, and a random effect of litter were included in these models. Kittens were explored as an additional random effect; however, it was removed as the missing datapoints impacted the variance estimation. There were some missing datapoints due to animal behaviour reasons. A total of 9.6% of planned blood sample collections were unsuccessful, and 34.1% of blood sample collections did not achieve the full volume required for all planned measures. All tables and figures provide sample size information. Mixed model methodology was utilised to account for the repeated measurements in each animal violating the assumption of independence. Assumptions of normality and constant variance were assessed through the visual inspection of residuals. If the assumptions were deemed to be violated, the response variable was log10 transformed; where this was not amenable, the response variable was rank transformed. In addition, the boxplots of the residuals were plotted to assess the assumption of homogeneity of variance across the diet and age groups. If this assumption was deemed to be violated, a weight was added to the variances by either the diet or age variable. Antibody titre data were log2 transformed prior to analysis [40]. Two sets of hypothesis tests were performed using an overall family wise error rate of 5%, i.e., the difference between diet group at each of the timepoints and the change from the earliest timepoint to each subsequent timepoint within each diet group. Data are presented as means with 95% confidence intervals (CI) or medians with inter quartile range (IQR) if rank transformed. A significant difference was determined with a *p*-value of ≤0.05.

A protective titre is recognised as ≥1:40 [38,41,42,43] and ≥1:8 [38] for FPV and FHV, respectively, and differences between diet groups in proportion of kittens reaching a protective titre following the complete course of vaccination at week 27 were analysed by a Fisher’s exact test. A significant difference was determined with a *p*-value of ≤0.05. A adequate response to vaccination is recognised as a four-fold titre increase [42,43], and fold changes were determined between the sample timepoints, following the complete course of vaccination (week 23 and 27) to all previous sample collection timepoints (week 10, 12, 14 and 18) for FPV and FHV. For each comparison, an adequate response was recorded if there was a four-fold titre increase [42,43]; otherwise, an inadequate response was recorded. Fisher’s exact test was used to compare the proportion of adequate responses for the diet groups between the paired timepoints. The resulting *p*-values were compared against the Bonferroni-corrected alpha value, 0.05/8 = 0.00625, to determine significance.

All analyses were performed using R v 3.6.3 software [44], using libraries lme4 [45] for linear mixed effects models and multcomp [46] for simultaneous estimation.

## 3. Results

All forty kittens completed the full duration of the study. All kittens remained healthy and did not display any clinically relevant signs of concern or adverse health related to diet intake at any point during the study.

### 3.1. Haematology and Serum Biochemistry

There were significant between-diet group differences (*p* < 0.05) at single timepoints for several haematology and biochemistry parameters, WBCs, lymphocytes, monocytes, haemoglobin, hematocrit, RBCs, total protein, calcium and potassium. Significant changes in haematology and biochemistry parameters (*p* < 0.05) were also observed within a diet group in a single or both diet groups (Table 2 and Supplementary Table S2).

Serum liver enzymes, ALT at 10, 14 and 18 weeks and AST at 12 and 18 weeks, were significantly (*p* < 0.05) lower in the test diet group by 20.1–35.5% compared to the control diet group. Furthermore, 15% and 6% of ALT and 24% and 12% of AST values exceeded the reference range reported for kittens [47] for control and test diet groups, respectively. Serum triglycerides at 10, 12 and 27 weeks and ALP at 12 and 18 weeks were significantly (*p* < 0.05) higher in the test diet group by 24.3–46.8% compared to the control diet group. All ALP values for all kittens, and most triglyceride values in both diet groups remained within reference range [47] (Table 2). 

### 3.2. Intake, Bodyweight, Body Condition Score and Physical Measurements 

Both diets were well accepted by the kittens throughout the study, and diet refusal was low, with 5.0% and 3.3% refusal for the control and test diet groups during the study, respectively. Intake (g) and energy intake (kcal) significantly (*p* < 0.05) increased from week 10 for both diet groups; however, they were significantly (*p* < 0.05) higher in the control diet group by 9.6–12.5% compared to the test diet group at weeks 23 and 27. Intake per metabolic BW (kcal per kg BW^0.67^) was significantly lower (*p* < 0.05) in the control diet group by 4.7–8.0% compared to the test diet group for up to 14 weeks. BW and BCS were not significantly different between the diet groups; however, significant (*p* < 0.05) increases during the course of the study were found for both diet groups (Table 3).

Height at withers and rump width were not significantly different between the diet groups. However, thoracic girth was significantly larger (Control: 29.3 (28.3, 30.3) cm; Test: 30.9 (30.2, 31.7) cm; *p* = 0.013) in the test diet group compared to the control diet group (Supplementary Table S3).

### 3.3. Serum Antibodies

**Figure 1:**
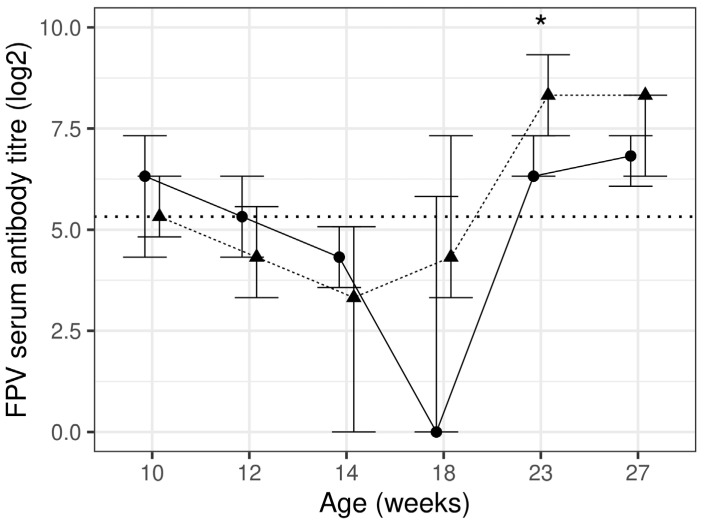
Feline parvovirus titre using haemagglutinin inhibition assay at each timepoint presented as median and inter-quartile range (IQR) for control (*n* 17–20; —●—) and test (*n* 15–20; ---▲---) diet groups. The horizontal dotted line represents the protective antibody titre level. * significant between diet group difference (*p* < 0.05).

The test diet group had a significantly (*p* = 0.023) higher antibody titre against FPV at week 23 by 31.7% when compared to the control diet group. Except for one control diet group kitten, all kittens had a detectable pre-vaccination antibodies titre against FPV at week 10 (>1:10). When compared to week 10, the antibody titre significantly (*p* < 0.05) declined at week 14 for both diet groups and significantly (*p* < 0.05) increased at week 23 and 27 in the test diet group only (Figure 1, Table 4). The test diet group had a higher percentage of kittens with higher titre levels (≥1:160) [43,48], following the complete course of vaccination, i.e., 83% and 71% of test compared to 42% and 50% of the control diet group at weeks 23 and 27, respectively. In total, 85% and 95% of kittens had an adequate response to vaccination in the control and test diet groups, respectively; however this difference did not reach a statistical significance (*p* > 0.00625). All kittens in the test diet group reached the protective titre level (≥1:40 [38,41,42,43]) at week 27 compared to 85% of kittens in the control diet group; however, this difference did not reach a statistical significance (*p* = 0.234). 

**Figure 2:**
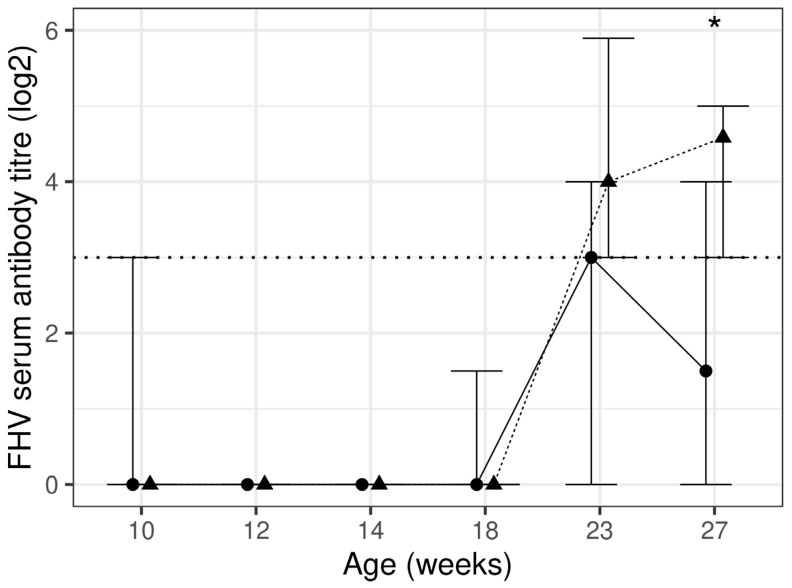
Feline herpesvirus titre using virus neutralisation assay at each timepoint presented as median and inter-quartile range (IQR) for control (*n* 16–20; —●—) and test (*n* 14–20; ---▲---) diet groups. The horizontal dotted line represents the protective antibody titre level. * significant between diet group difference (*p* < 0.05).

The test diet group had a significantly (*p* = 0.007) higher antibody titre against FHV at week 27, by 205%, when compared to the control diet group. A total of seven kittens (six from the control and one from the test diet group) had a detectable pre-vaccination antibody titre against FHV at week 10 (>1:8). The antibody titre significantly (*p* < 0.001) increased at week 23 and 27 from week 10 in the test diet group only (Figure 2, Table 4). The test diet group had a higher percentage of kittens with higher titre levels (≥1:32), following the complete course of vaccination, i.e., 39% and 41% compared to 21% and 5% for the control diet group at weeks 23 and 27, respectively. In total, 20% and 35% of kittens had an adequate response to vaccination for control and test diet group kittens, respectively. There was a significant (*p =* 0.005) between diet group difference for adequate response to vaccination when comparing the titre at 27 weeks with the one at 18 weeks. Overall, 50% and 88% of kittens reached the protective titre level (≥1:8 [38]) at week 27 in the control and test diet groups, respectively, and this difference was significant (*p* = 0.017). 

**Figure 3:**
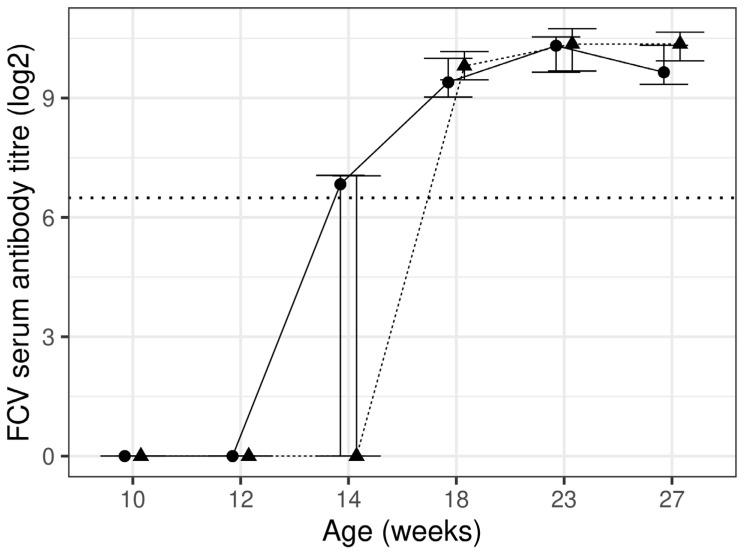
Feline calicivirus titre using ELISA assay at each timepoint presented as median and inter-quartile range (IQR) for control (*n* 15–19; —●—) and test (*n* 13–20; ---▲---) diet groups. The horizontal dotted line represents the protective antibody titre level.

There was no significant difference between the diet groups for antibody titre against FCV; however, significant (*p* < 0.05) increases during the course of the study were found for both diet groups (Figure 3, Table 4). No kitten had an antibody titre at protective levels (≥90, laboratory reference range) in either diet group at week 10. Protective levels were reached for 12% and 21% of kittens at week 12 and 64% and 41% of kittens at week 14 for the control and test diet groups, respectively. All kittens in both diet groups reached protective levels at week 18, and this was maintained for the remainder of the study.

Serum IgM was significantly (*p* < 0.05) higher at all timepoints in the test diet group, by 3.9–30.5%, when compared to the control diet group. There were no significant differences between diet groups for IgA or IgG. All IGs significantly (*p* < 0.05) increased over time in both diet groups (Table 4).

### 3.4. Immune Function Assays

There were no significant differences between diet groups in Con A- and SEB-stimulated lymphocyte proliferation, although significant (*p* < 0.05) increases from week 12 were found for SEB-stimulated lymphocyte proliferation at both EC50 and EC80 in the test diet group only. PMA/ION (EC80 only)-stimulated lymphocyte proliferation was significantly (*p* = 0.046) lower in the test diet group at week 12 compared to the control diet group; however, proliferation significantly (*p* < 0.05) increased at weeks 18 and 27 in the test diet group and was no longer significantly different from the control diet group. There were no other significant differences found at either concentration or any other timepoints (Supplementary Table S4).

There was no significant between diet group difference in the proportion of T-cells (CD5+), T-helper cells (CD5+4+), cytotoxic T-cells (CD5+8+), B-lymphocytes (CD21+), granulocytes (CD18+) and T-helper cells: cytotoxic T-cells (CD5+CD4+: CD5+CD8+). The proportion of cytotoxic T-cells significantly (*p* < 0.05) increased and T-helper cells: cytotoxic T-cells significantly (*p* < 0.05) decreased between weeks 18 and 27 in both diet groups (Supplementary Tables S4 and S5).

There was no significant difference between diet group or over time within diet group for phagocytosis (Supplementary Table S4).

### 3.5. Serum Cytokines

GM-CSF and IL-1 β were significantly higher (*p* < 0.05) in the test diet group compared to the control diet group at a single timepoint, and no other between diet group differences were found. Significant (*p* < 0.05) changes within diet group during the course of the study in a single or both diet groups were found for several cytokines (Supplementary Table S6).

### 3.6. Nucleotides

The primary method used to quantify extracted nucleotides found that uridine nucleotides were significantly (*p* < 0.05) lower in the test diet group when compared to the control diet group at 18 and 27 weeks. There were no significant between diet group differences for guanosine and adenosine nucleotides. Uridine and guanosine nucleotides significantly (*p* < 0.05) decreased over the course of the study in both diet groups. The secondary method, which measured nucleosides formed by the dephosphorylation of the nucleotides, found that uridine and cytosine nucleosides were significantly (*p* < 0.05) lower in the test diet group when compared to the control diet group at all timepoints. There were no significant between diet group differences for guanosine and adenosine nucleosides; however, guanosine nucleosides significantly (*p* < 0.05) decreased over the course of the study in both diet groups (Supplementary Table S7).

## 4. Discussion

The purpose of this study was to determine if a diet supplemented with nucleotides, scFOS, XOS, β-carotene and vitamin E could positively influence health and immune parameters when fed to domestic short hair kittens until 28 weeks of age. 

There is no single biomarker that can define immune status, function or its relationship with nutrition; therefore, multiple parameters were measured to assess the different aspects of the immune system. Vaccination causes a coordinated and integrated immune response, providing the host with protection against disease and represents a response similar to a natural infection, and *in vivo* vaccine challenge is recognised as a clinically relevant measure of immune function [49]. In total, 97%, 23% and 0% of kittens had a detectable pre-vaccination antibody titre against FPV, FHV and FCV, respectively. Pre-vaccination antibodies, in the absence of clinical symptoms of infection, are likely maternally derived antibodies (MDA), mostly acquired through nursing [50]. MDAs have been reported to decrease to undetectable levels between 6–8 weeks for FHV [51], 10–14 weeks for FCV [52] and 8–20 weeks for FPV [36,50]; however, our data demonstrate that antibodies against FHV can persist until 12 weeks in some kittens. For most kittens who had pre-vaccination antibodies, antibodies decreased to below protective levels by 14 weeks for FHV and 18 weeks for FPV. The antibody titre increasing at further timepoints likely demonstrates that kittens seroconvert in response to the course of vaccination. Our data largely demonstrate seroconversion occurring 8 weeks following the first dose of vaccination for FHV and FPV. We find the response against FCV was quicker, and seroconversion was observed from 2 weeks following the first dose of vaccination; however, the analysis was completed using a sensitive ELISA method rather than a functional antibody assay. Seroconversion, seven days post-vaccination, has been reported in kittens; however, vaccines, vaccination schedules and analysis methods differ [53].

The vaccine response data in our study find that the median antibody titre was significantly greater in the test diet group by 31.7% for FPV and 205% for FHV. Furthermore, 10% more kittens in the test diet group demonstrated an adequate response to the FPV vaccination and 15% more kittens in the test diet group demonstrated an adequate response to the FHV vaccination when compared to the control group. All kittens in the test diet group were protected against FPV compared to 85% in the control diet group, and 88% of kittens in the test diet group were protected against FHV compared to 50% in the control diet group. Therefore, our data find that the test diet group had a stronger humoral immune response to an *in vivo* vaccine immune challenge with higher antibody levels, a greater vaccine responsiveness and a higher proportion of kittens protected following the complete course of vaccination. The presence of antibodies following a vaccination is associated with resistance against infection [50,54]; therefore, test diet group kittens in this study may have a greater defence capacity against infectious disease than the control group. Our results are in agreement with other studies where diets containing nucleotides produced improved responses to vaccines [6,8,9,10].

Studies in cats have reported a similar level of protection or vaccine response against FPV, FHV and FCV as the present study [38,42,53,55,56]; however, there are some contradictory findings for FHV. One study found 8.3% of cats had an adequate response to FHV vaccination [37] whilst another study reports a 88.2% adequate response rate [42]. Inactivated vaccines for FHV (as used in the present study) have been shown to produce higher antibody levels when compared to modified live vaccines [38], and a study using an inactivated vaccine found all cats developed neutralising antibodies against FHV and FPV [56]; however, this response level was not found in the present study. There are difficulties in making direct comparisons to other studies, owing to a lack of method standardisation. Furthermore, vaccine type, antigen strain, dose, adjuvants, age, genetics, vaccination history, environment, lifestyle and breed are factors that impact vaccine responsiveness [36,37,38,43]. 

Nine kittens from control (including four littermates from a litter of four and three littermates from a litter of four) and two kittens from test diet group did not seroconvert against FHV following vaccination; however, only six of these kittens (including two of the four littermates and one of the three littermates) had detectable pre-vaccination antibodies at week 10. Only one kitten from a total of seven, who had a detectable pre-vaccination FHV antibody titre, seroconverted and reached a protective antibody titre during the study. This suggests MDAs and genetics are factors that can impact vaccine responsiveness. Three control diet group littermates (from a litter of four) did not seroconvert against FPV following vaccination; interestingly, these kittens were among seven kittens who had a high pre-vaccination antibody titre at week 10 (≥1:160) [43,48], which has been linked to poor vaccine response [43]. Whilst this again may support that high pre-vaccination antibody levels may interfere with response to vaccination [36], it should be noted that the remaining four kittens (including one from the same litter) demonstrated an adequate response to vaccination and reached a protective antibody titre; therefore, other factors such as diet maybe influential in modulating the vaccine antigen immune response. 

The test diet group kittens had higher circulating serum IgM throughout the study compared to the control group. IgM is the subclass of IGs, which are produced early following antigen exposure [57]; thus, its elevated levels may indicate an active infection or response to vaccination, and its low levels may be linked to a higher risk of infections [58]. These results are in agreement with nucleotides supplementation studies in preterm infants and beagle puppies [8,12] and suggest the test diet induces a greater antibody production, which in turn enhances the humoral immune defence against invading pathogens.

Vaccination would also likely induce a cell-mediated immune response, especially against viruses that become intracellular such as FHV [59]. Studies have demonstrated increased lymphocyte proliferation, phagocytic activity and cytokines expression with dietary nucleotide supplementation [8,10,13], whilst the methods used differ, the present study did not find an increase in lymphocyte proliferation and phagocytic activity. The proinflammatory cytokine IL-1β was increased at a single timepoint in the test diet group, which may suggest a greater innate response to vaccination; alternatively, this may be a transient difference unrelated to diet. No differences between diet group were found for any cell population during this study, however, additional statistical analysis comparing diet group irrespective of age revealed significantly (*p* < 0.05) higher proportion of T-helper cells in the test diet group. T-helper cells activate B-cells that subsequently produce antibodies. The possible higher proportion of these cells and the stronger humoral immune response to vaccination maybe linked; however, this would require further investigation. This study was only statistically powered for the primary measure; therefore, further research using appropriately powered studies is required to truly assess the dietary impact on other measures.

The present study investigated a cocktail of ingredients; therefore, it is not known which component or synergistic effects are driving the between diet group differences that were found. Further studies would be required to determine which individual component or combination is driving the results found in this study. Whilst the mechanisms are not fully understood, there is compelling evidence that the provision of dietary nucleotides has a favourable effect on humoral immune responses to vaccination in many species during growth including cats, dogs and humans [6,8,10,60]. Nucleotides may have a direct effect on immune cells by becoming incorporated into cells to form ATP [61] or increasing DNA synthesis as well as influencing maturation, activation and proliferation [62]. Different nucleotides have been found to enhance the different aspects of immune function, which suggests they are immunomodulators for different defence mechanisms [63]. Dietary β-carotene and vitamin E have also been reported to enhance humoral immune response to immune challenge in dog [30,64,65]; therefore, it is likely that nucleotides, β-carotene and vitamin E play a role in humoral immunity. The test diet components may also have indirect effects on immune health through the microbiome as nucleotides [66], scFOS [17] and XOS [22,23] have been associated with improving the gut microbiome and increasing bacterial populations such as bifidobacterium and lactobacilli, which are reported to exert positive effects on immune function [18]. In humans, infants fed a scFOS-supplemented formula had increased faecal bifidobacteria and higher faecal poliovirus-specific IgA following vaccination [67]. Studying the gut microbiome of kittens fed the test diet would require further work; however, it would provide insights on how the microbiome may influence the immune system. 

The higher diet intake (g/day and kcal/day) in the control diet group at week 23 and week 27 appears to be driven by five maternally related control diet group kittens from two litters. These kittens had higher intakes relative to all other kittens on the study and were among the heaviest on the study. The intake of the test diet group had much less between kitten variation compared to the control diet group, and the intake was at a similar level to the remaining 15 kittens in the control diet group. The amount of diet offered to kittens was individual, based on amounts to maintain an ideal BW and BCS, which did not differ between diet groups. When intake was adjusted for metabolic BW (kcal/per kg BW^0.67^), there was no significant between diet group difference at 23 and 27 weeks. Interestingly, the test diet group did have a significantly higher intake kcal/per kg BW^0.67^ for up to 14 weeks. The reason for these differences is unclear; however, a higher intake variation in the early weeks, diet palatability, the effect of litter, genetics and weaning age may be contributing factors. Thoracic girth was larger for kittens in the test diet group, and nucleotides have been associated with growth in humans [68] based on morphometric measurements and weight; therefore, this finding suggests that nucleotides may support growth during early life. 

It may be expected that nucleotide supplementation increases the blood pool of nucleotides, nucleosides and free bases. Dietary nucleotides are understood to be broken down before absorption, and an *in vivo* study found that exogenous nucleosides increase the intracellular concentrations of UTP [69] and therefore could impact extracellular or blood concentrations. Our study found that whole blood uridine and cytosine nucleotides are lower in the test diet group. Although counter-intuitive, this could suggest a better utilisation of the nucleotides in the test diet group. The reasons and mechanisms are not fully understood; however, it should be noted that blood nucleotides were determined from fasted animals and that post-prandial samples taken shortly after feeding may have revealed different results.

Serum biochemistry and haematology are measures of systemic health, and some parameters revealed differences between diet groups; however, they do not indicate a adverse effect to health. Reference ranges are useful to assess if a result is within the expected range for a healthy animal; however, they may not be widely available or up to date for growing animals where rapid changes occur during the early weeks. In the present study, the liver enzymes ALT and AST were lower in the test diet group for up to 18 weeks of age, however this is unlikely to be of concern as adverse health effects are associated with elevated enzyme levels. Some ALT and AST results exceeded the reference range [47] for some kittens, mostly at a single timepoint, and a majority of these were from the control diet group and therefore unlikely related to the additional ingredients in the test diet. 

Although within reference range [47], a transient elevation in ALP was found between 12 to 18 weeks of age in the test diet group. One test diet group litter of six kittens had higher ALP levels relative to the rest of the test diet group at early timepoints, which may be driving the difference between diet group. We did not observe elevations for liver enzymes ALT and AST in the test diet group, and there were no reports of clinically relevant symptoms to suggest adverse health effects; therefore, these differences are unlikely to be clinically relevant. ALP isoenzymes are also expressed in bones, and we find an increased thoracic girth in test diet group kittens; therefore, the elevation of ALP may be related to growth; additional work to measure specific ALP isoenzymes such as a bone-specific ALP would be required to confirm this explanation. Triglycerides were higher in the test diet group at multiple timepoints during the course of the study with most values within the laboratory reference range. Although the reasons for differences in ALP and triglycerides are not fully understood, they are likely unrelated given the kittens with higher ALP levels had moderate triglyceride levels and vice versa; however, they could be linked to the diets fed in this study. Higher triglycerides and ALP have been reported in dogs fed extruded dry diets compared to lightly cooked grain free and raw diets [70]. The diets in the present study used the same core recipe; however, some processing differences did exist to enable the inclusion of the additional ingredients in the test diet. There is evidence that increased dietary carbohydrates may increase serum triglycerides [71]; however, the evidence is not conclusive [72]. In cats, a study found that XOS altered the gut microbiome, increasing the abundance of the genera *Collinsella* [73], which may increase triglyceride synthesis [74]. In humans, nucleotide supplementation resulted in higher triglycerides [75]; however, another study found no difference, although differences in other lipoproteins were determined [76]. Differences in ALP and triglycerides may simply reflect the variation in metabolism owing to the differences in the diets. 

Healthy kittens enrolled in the present study were assigned to a diet group in litters at birth; therefore, the diet groups could only be balanced to a certain extent. Although no two litters shared the same parents, the 13 litters enrolled on this study were bred from a total of ten queens and five tom cats; therefore, genetic links between different litters and between diet groups were inevitable. A completely balanced and randomised study would completely rule out variables related to litter and genetics. Between diet group differences beyond 28 weeks or the lasting effects of feeding these diets during growth on health and immune function in later life are unknown and are yet to be further investigated.

## 5. Conclusions

This study provides evidence that kittens fed a diet containing nucleotides, scFOS, XOS, β-carotene and higher levels of vitamin E had a stronger humoral immune response to vaccination, resulting in higher adequate seroconversion rates and higher number of kittens reaching the protective thresholds compared to a control group. This suggest that the test diet supports immune defence, enabling a greater response to immune challenges.

## Figures and Tables

**Table 1:** Nutrient composition of the control and test diets. Diet analysis was performed at Eurofins Ltd. The values presented in this table are averages and are within conformance standards.

	Control Batch 1	Control Batch 2	Test
Dry matter (g/100 g)	94.08	94.80	94.35
Moisture (g/100 g)	5.92	5.20	5.65
Protein (g/100 g)	32.00	32.60	30.90
Fat (g/100 g)	18.00	17.30	18.20
Ash (g/100 g)	6.50	7.10	6.70
Calcium (g/4184 kJ (1000 kcal))	3.06	3.44	3.10
Phosphorus (g/4184 kJ (1000 kcal))	2.38	2.53	2.46
Calcium: Phosphorus ratio	1.29	1.36	1.26
Sodium (g/4184 kJ (1000 kcal))	1.04	1.24	1.22
Chloride (g/4184 kJ (1000 kcal))	2.12	2.29	2.04
Potassium (g/4184 kJ (1000 kcal))	1.63	1.73	1.84
Magnesium (mg/4184 kJ (1000 kcal))	213.00	248.37	231.00
Iron (mg/4184 kJ (1000 kcal))	39.73	45.74	45.25
Copper (mg/4184 kJ (1000 kcal))	3.46	3.30	3.61
Manganese (mg/4184 kJ (1000 kcal))	16.07	21.17	15.76
Zinc (mg/4184 kJ (1000 kcal))	42.70	39.84	35.41
Iodine (mg/4184 kJ (1000 kcal))	1.15	1.00	1.05
Selenium (µg/4184 kJ (1000 kcal))	99.46	88.53	97.13
Vitamin A (µg/4184 kJ (µg/1000 kcal))	2317	2228	2700
Vitamin D3 (µg/4184 kJ (µg/1000 kcal))	6.93	7.87	7.75
Vitamin E (mg/4184 kJ (mg/1000 kcal))	49.80	57.50	109.60
Thiamin (mg/4184 kJ (1000 kcal))	6.79	3.32	5.63
Riboflavin (mg/4184 kJ (1000 kcal))	12.64	14.66	12.07
Pantothenic acid (mg/4184 kJ (1000 kcal))	23.10	25.08	21.84
Niacin/nicotinic acid (mg/4184 kJ (1000 kcal))	74.53	45.25	59.51
Pyridoxine (mg/4184 kJ (1000 kcal))	6.94	5.88	5.61
Folic acid (µg/4184 kJ (1000 kcal))	2142	1898	1591
Cyanocobalamin (µg/4184 kJ (1000 kcal))	52.81	51.15	45.41
Choline (mg/4184 kJ (1000 kcal))	548	492	590
Linoleic acid (g/4184 kJ (1000 kcal))	8.72	8.68	8.97
Alpha-Linoleic acid (g/4184 kJ (1000 kcal))	0.91	0.73	0.81
Arachidonic acid (g/4184 kJ (1000 kcal))	0.23	0.21	0.23
Eicosapentenoic acid (g/4184 kJ (1000 kcal))	0.16	0.14	0.13
Docosahexaenoic acid (g/4184 kJ (1000 kcal))	0.10	0.10	0.10
Arginine (g/4184 kJ (1000 kcal))	4.12	4.38	3.89
Cystine (g/4184 kJ (1000 kcal))	1.08	1.13	0.96
Histidine (g/4184 kJ (1000 kcal))	1.49	1.57	1.46
Taurine (mg/4184 kJ (1000 kcal))	520.75	390.99	523.77
Isoleucine (g/4184 kJ (1000 kcal))	2.71	2.83	2.53
Leucine (g/4184 kJ (1000 kcal))	5.28	5.63	5.07
Lysine (g/4184 kJ (1000 kcal))	4.54	4.84	4.70
Methionine (g/4184 kJ (1000 kcal))	1.96	1.84	1.88
Phenylalanine (g/4184 kJ (1000 kcal))	3.26	3.37	2.98
Threonine (g/4184 kJ (1000 kcal))	2.64	2.75	2.61
Tryptophan (g/4184 kJ (1000 kcal))	0.73	0.71	0.61
Tyrosine (g/4184 kJ (1000 kcal))	2.14	2.24	1.97
Valine (g/4184 kJ (1000 kcal))	3.36	3.64	3.20
Alanine (g/4184 kJ (1000 kcal))	4.07	4.21	3.87
Aspartic acid (g/4184 kJ (1000 kcal))	5.11	5.21	5.01
Glutamic acid (g/4184 kJ (1000 kcal))	15.40	15.91	14.46
Glycine (g/4184 kJ (1000 kcal))	5.65	5.85	5.36
Proline (g/4184 kJ (1000 kcal))	6.17	6.69	5.68
Serine (g/4184 kJ (1000 kcal))	3.53	3.84	3.38

**Table 2:** Serum biochemistry. Serum biochemistry values are provided as means and 95% confidence intervals (CI) for control and test kitten diet groups. *p* values represent comparison between diet group at the sample collection timepoint (*p* < 0.05 is statistically significant).

Parameter	Weeks	Control			Test			*p*
		*n*	Mean	95% CI	*n*	Mean	95% CI	
Total protein (g/L)	10	15	53.72	52.40, 55.04	14	54.16	52.70, 55.62	0.649
	12	13	54.72	53.37, 56.07	15	56.84 *	55.39, 58.30	0.037
	14	13	55.04	53.67, 56.42	15	56.91 *	55.47, 58.35	0.065
	18	18	58.99 *	57.75, 60.24	11	59.53 *	57.97, 61.09	0.587
	23	17	61.51 *	60.23, 62.78	17	62.80 *	61.40, 64.21	0.171
	27	18	62.23 *	60.99, 63.48	14	62.69 *	61.23, 64.14	0.632
Albumin (g/L)	10	15	30.93	30.03, 31.82	14	30.41	29.39, 31.43	0.439
	12	13	30.55	29.64, 31.47	15	31.08	30.06, 32.09	0.435
	14	13	31.16	30.24, 32.09	15	30.96	29.95, 31.97	0.763
	18	18	32.44 *	31.57, 33.30	11	32.54 *	31.48, 33.61	0.875
	23	17	32.36 *	31.48, 33.24	17	32.11 *	31.11, 33.10	0.699
	27	18	33.18 *	32.32, 34.05	14	33.35*	32.34, 34.37	0.792
Glucose (mmol/L)	10	15	6.01	5.75, 6.29	14	5.66	5.39, 5.95	0.074
	12	13	5.37 *	5.13, 5.62	15	5.34	5.08, 5.61	0.883
	14	13	5.23 *	4.997, 5.48	15	5.19 *	4.95, 5.45	0.811
	18	18	5.04 *	4.83, 5.25	11	5.02 *	4.76, 5.29	0.898
	23	17	4.89 *	4.68, 5.10	17	4.84 *	4.61, 5.07	0.754
	27	18	4.87 *	4.67, 5.08	14	4.82 *	4.59, 5.06	0.738
Inorganic phosphorus (mmol/L)	10	15	2.70	2.59, 2.81	14	2.66	2.54, 2.79	0.660
	12	13	2.53 *	2.42, 2.64	15	2.53	2.41, 2.66	0.997
	14	13	2.62	2.51, 2.74	15	2.54	2.41, 2.66	0.311
	18	18	2.50 *	2.40, 2.61	11	2.42 *	2.29, 2.55	0.285
	23	17	2.35 *	2.25, 2.46	17	2.35 *	2.22, 2.47	0.911
	27	18	2.22 *	2.12, 2.33	14	2.07 *	1.95, 2.20	0.074
Alanine aminotransferase (U/L)	10	15	48.28	39.69, 58.72	14	35.12	28.11, 43.87	0.037
	12	13	45.70	37.46, 55.75	15	35.77	28.63, 44.67	0.102
	14	13	53.28	43.59, 65.12	15	34.38	27.58, 42.86	0.006
	18	18	52.51	43.49, 63.41	11	37.65	29.87, 47.46	0.031
	23	17	51.07	42.16, 61.87	17	39.90	32.10, 49.59	0.090
	27	18	51.85	42.93, 62.62	14	44.40	35.58, 55.42	0.279
Aspartate aminotransferase (U/L)	10	15	22.47	19.78, 25.17	14	20.07	17.11, 23.03	0.231
	12	13	23.64	20.86, 26.42	15	18.88	15.93, 21.83	0.022
	14	13	20.88	18.06, 23.71	15	18.91	16.01, 21.81	0.330
	18	18	22.41	19.90, 24.93	11	16.84	13.65, 20.04	0.008
	23	17	20.19	17.59, 22.78	17	18.28	15.46, 21.10	0.318
	27	18	21.19	18.66, 23.71	14	18.39	15.46, 21.33	0.152
Calcium (mmol/L)	10	15	2.65	2.61, 2.69	14	2.66	2.61, 2.70	0.840
	12	13	2.58 *	2.54, 2.62	15	2.64	2.60, 2.68	0.043
	14	13	2.63	2.59, 2.67	15	2.67	2.63, 2.71	0.198
	18	18	2.62	2.58, 2.65	11	2.65	2.61, 2.70	0.192
	23	17	2.60	2.56, 2.63	17	2.63	2.59, 2.67	0.236
	27	18	2.55 *	2.52, 2.59	14	2.58 *	2.54, 2.62	0.352
Cholesterol (mmol/L)	10	15	4.11	3.58, 4.65	14	4.49	3.87, 5.11	0.344
	12	13	3.88	3.33, 4.42	15	4.41	3.79, 5.04	0.184
	14	13	3.76	3.22, 4.30	15	4.34	3.72, 4.96	0.152
	18	18	3.81	3.28, 4.34	11	4.46	3.83, 5.10	0.113
	23	17	3.62 *	3.08, 4.15	17	4.30	3.68, 4.91	0.093
	27	18	3.71	3.18, 4.24	14	4.20	3.58, 4.82	0.218
Urea (mmol/L)	10	15	6.92	6.08, 7.76	14	8.08	7.11, 9.05	0.074
	12	13	7.29	6.44, 8.13	15	8.25	7.28, 9.22	0.132
	14	13	7.10	6.25, 7.95	15	7.63	6.66, 8.60	0.396
	18	18	7.63	6.81, 8.45	11	7.96	6.96, 8.95	0.598
	23	17	7.79 *	6.96, 8.62	17	8.01	7.04, 8.97	0.721
	27	18	7.36	6.53, 8.18	14	7.46	6.49, 8.43	0.868
Triglycerides (mmol/L)	10	15	0.38	0.32, 0.45	14	0.50	0.41, 0.60	0.036
	12	13	0.40	0.34, 0.48	15	0.59	0.49, 0.71	0.005
	14	13	0.48	0.40, 0.57	15	0.58	0.48, 0.70	0.139
	18	18	0.45	0.39, 0.53	11	0.49	0.40, 0.60	0.606
	23	17	0.43	0.37, 0.51	17	0.53	0.45, 0.64	0.097
	27	18	0.45	0.38, 0.53	14	0.63	0.52, 0.76	0.009
Alkaline phosphatase (U/L)	10	15	121.5	100.3, 142.7	14	142.8	118.7, 166.9	0.181
	12	13	133.8	112.3, 155.3	15	166.3	142.2, 190.4	0.048
	14	13	127.9	106.2, 149.6	15	158.9	135.0, 182.8	0.059
	18	18	120.0	99.6, 140.4	11	154.4	129.3, 179.5	0.038
	23	17	102.4	81.6, 123.2	17	120.6	97.0, 144.2	0.239
	27	18	92.0 *	71.6, 112.5	14	119.0	95.0, 143.0	0.089
Creatinine (µmol/L)	10	15	58.57	52.51, 64.63	14	56.47	49.49, 63.45	0.640
	12	13	60.08	53.94, 66.22	15	58.40	51.47, 65.34	0.710
	14	13	64.40	58.21, 70.59	15	59.31	52.43, 66.19	0.264
	18	18	73.48 *	67.61, 79.35	11	73.25 *	66.08, 80.42	0.959
	23	17	86.12 *	80.16, 92.08	17	85.35 *	78.55, 92.16	0.860
	27	18	91.66 *	85.78, 97.54	14	92.35 *	85.44, 99.26	0.874
Sodium (mmol/L)	10	15	147.7	147.0, 148.4	14	147.1	146.4, 147.9	0.298
	12	13	148.0	147.3, 148.7	15	147.1	146.3, 147.9	0.100
	14	13	148.1	147.4, 148.9	15	148.1	147.3, 148.9	0.964
	18	18	149.0 *	148.3, 149.7	11	149.6 *	148.8, 150.5	0.240
	23	17	149.7 *	149.0, 150.4	17	149.8 *	149.0, 150.5	0.826
	27	18	150.0 *	149.4, 150.7	14	150.0 *	149.3, 150.8	0.985
Potassium (mmol/L)	10	15	5.12	4.97, 5.26	14	5.04	4.89, 5.19	0.468
	12	13	4.82 *	4.66, 4.97	15	5.04	4.89, 5.19	0.037
	14	13	4.87	4.72, 5.03	15	5.02	4.88, 5.17	0.166
	18	18	4.70 *	4.54, 4.83	11	4.79	4.62, 4.96	0.397
	23	17	4.63 *	4.49, 4.76	17	4.60 *	4.46, 4.74	0.747
	27	18	4.53 *	4.40, 4.66	14	4.46 *	4.31, 4.62	0.537
Chloride (mmol/L)	10	15	115.5	114.8, 116.3	14	115.6	114.7, 116.4	0.935
	12	13	115.4	114.6, 116.2	15	115.1	114.3, 116.0	0.620
	14	13	116.0	115.2, 116.8	15	115.0	114.2, 115.8	0.084
	18	18	115.7	115.0, 116.4	11	116.3	115.3, 117.2	0.318
	23	17	116.9 *	116.1, 117.6	17	116.0	115.1, 116.8	0.098
	27	18	116.8 *	116.0, 117.5	14	116.3	115.4, 117.1	0.384
Globulin (g/L)	10	15	22.81	21.65, 23.97	14	23.77	22.49, 25.05	0.268
	12	13	24.18	22.99, 25.37	15	25.80 *	24.52, 27.07	0.068
	14	13	23.89	22.68, 25.10	15	25.96 *	24.70, 27.22	0.022
	18	18	26.56 *	25.47, 27.65	11	27.02 *	25.65, 28.40	0.592
	23	17	29.16 *	28.04, 30.28	17	30.71 *	29.48, 31.94	0.066
	27	18	29.07 *	27.98, 30.16	14	29.35 *	28.07, 30.62	0.740

* A significant difference from week 10 within diet group (*p* < 0.05).

**Table 3:** Intake, bodyweight and body condition score. Intake, bodyweight and body condition score values are provided as medians and interquartile range (IQR) for control and test kitten diet groups. *p* values represent comparison between the diet groups at the sample collection timepoint (*p* < 0.05 is statistically significant).

Parameter	Control				Test			*p*
	Week	*n*	Median	IQR	*n*	Median	IQR	
Diet intake (g/day)	10	20	44	33, 51	20	46	37, 51	0.852
	12	20	55 *	52, 59	20	57 *	55, 60	0.134
	14	20	60 *	55, 66	20	64 *	58, 67	0.207
	18	20	65 *	57, 68	20	62 *	57, 66	0.702
	23	20	65 *	61, 68	20	59 *	57, 65	0.021
	27	20	63 *	55, 70	20	56 *	54, 63	0.027
Calorie intake (kcal/day)	10	20	177	134, 206	20	187	149, 207	0.807
	12	20	223 *	211, 239	20	232 *	224, 244	0.101
	14	20	243 *	221, 267	20	260 *	236, 272	0.160
	18	20	263 *	231, 276	20	252 *	232, 268	0.862
	23	20	263 *	248, 276	20	240 *	231, 264	0.029
	27	20	256 *	223, 285	20	228 *	220, 256	0.037
Calorie intake per metabolic bodyweight (kcal/kg bodyweight^0.67^)	10	20	150	113, 179	20	163	130, 180	0.043
	12	20	165 *	154, 176	20	174 *	166, 182	0.018
	14	20	164 *	150, 172	20	172 *	160, 183	0.016
	18	20	143 *	132, 150	20	141 *	130, 148	0.517
	23	20	126 *	121, 135	20	120 *	115, 125	0.093
	27	20	114 *	107, 121	20	106 *	102, 114	0.205
Body condition score	10	20	5	5, 5	20	5	5, 5	0.998
	12	20	5	5, 5	20	5	5, 5	0.837
	14	20	5	5, 5	20	5	5, 6	0.313
	18	20	5 *	5, 6	20	6 *	6, 6	0.077
	23	20	5 *	5, 6	20	6 *	5, 6	0.219
	27	20	6 *	5, 6	20	6 *	6, 6	0.053
Bodyweight (g)	10	20	1320	1200, 1407	20	1247	1165, 1321	0.494
	12	20	1599 *	1467, 1704	20	1537 *	1441, 1624	0.412
	14	20	1917 *	1736, 2018	20	1816 *	1712, 1932	0.441
	18	20	2514 *	2215, 2596	20	2365 *	2240, 2531	0.461
	23	20	3063 *	2631, 3211	20	2845 *	2687, 3099	0.414
	27	20	3401 *	2927, 3674	20	3193 *	2992, 3458	0.468

* A significant difference from week 10 within diet group (*p* < 0.05).

**Table 4:** Serum antibody titres and immunoglobulins. Serum antibody titres and immunoglobulin values are provided as median and inter-quartile range (IQR) for control and test diet kitten group. *p* values represent the comparison between the diet groups at the sample collection timepoint (*p* < 0.05 is statistically significant).

Parameter	Week	Control			Test			*p*
		* n *	Median	IQR	* n *	Median	IQR	
Calicivirus titre (log2)	10	16	0.00	0.00, 0.00	13	0.00	0.00, 0.00	0.726
	12	17	0.00	0.00, 0.00	20	0.00	0.00, 0.00	0.989
	14	15	6.83 *	0.00, 7.06	17	0.00	0.00, 7.04	0.29
	18	19	9.40 *	9.02, 10.00	18	9.80 *	9.46, 10.20	0.712
	23	18	10.30 *	9.65, 10.50	17	10.40 *	9.68, 10.70	0.809
	27	19	9.65 *	9.34, 10.30	16	10.40 *	9.93, 10.70	0.166
Herpesvirus titre (log2)	10	16	0.00	0.00, 3.00	14	0.00	0.00, 0.00	0.088
	12	19	0.00	0.00, 0.00	20	0.00	0.00, 0.00	0.659
	14	18	0.00	0.00, 0.00	18	0.00	0.00, 0.00	0.860
	18	19	0.00	0.00, 1.50	19	0.00	0.00, 0.00	0.317
	23	19	3.00	0.00, 4.00	18	4.00 *	3.00, 5.90	0.051
	27	20	1.50	0.00, 4.00	17	4.58 *	3.00, 5.00	0.007
Parvovirus titre (log2)	10	17	6.32	4.32, 7.32	15	5.32	4.82, 6.32	0.804
	12	19	5.32	4.32, 6.32	20	4.32	3.32, 5.57	0.398
	14	18	4.32 *	3.57, 5.07	18	3.32 *	0.00, 5.07	0.640
	18	19	0.00	0.00, 5.82	19	4.32	3.32, 7.32	0.144
	23	19	6.32	6.32, 7.32	18	8.32 *	7.32, 9.32	0.023
	27	20	6.82	6.07, 7.32	17	8.32 *	6.32, 8.32	0.064
IgA (µg/mL)	10	12	38.5	33.1, 51.7	12	81.8	32.1, 177.0	0.102
	12	15	56.1	49.3, 134.0	18	90.7	59.2, 178.0	0.145
	14	15	82.9 *	57.9, 144.0	17	81.1	61.8, 184.0	0.466
	18	19	142.0 *	89.7, 194.0	14	144.0 *	70.7, 318.0	0.560
	23	18	220.0 *	161.0, 280.0	17	146.0 *	74.4, 281.0	0.631
	27	17	205.0 *	92.1, 315.0	14	201.0 *	119.0, 274.0	0.470
IgG (µg/mL)	10	12	2435	2124, 2805	12	2406	1813, 2830	0.849
	12	15	3332 *	2656, 4300	18	4028 *	3454, 5663	0.360
	14	15	3464 *	2731, 4260	17	4131 *	3618, 6773	0.155
	18	19	5436 *	4871, 6465	14	6296 *	5140, 8668	0.888
	23	18	7574 *	6062, 8836	17	9019 *	6337, 11014	0.476
	27	18	7378 *	6142, 9150	14	9039 *	6032, 11900	0.653
IgM (µg/mL)	10	12	509	417, 699	12	625	539, 663	0.006
	12	15	709 *	486, 866	18	737 *	669, 848	0.000
	14	15	616	477, 779	17	673 *	624, 796	0.000
	18	19	620 *	508, 787	14	769 *	695, 920	0.000
	23	18	829 *	713, 1005	17	972 *	762, 1110	0.000
	27	18	752 *	662, 902	14	981 *	907, 1063	0.000

* A significant difference from week 10 within diet group (*p* < 0.05).

## Data Availability

All additional data are provided as Supplementary Material to this article.

## Supplementary Materials

The following supporting information can be downloaded at: https://www.mdpi.com/article/10.3390/ani13233734/s1, Table S1: Sample collection volumes and blood tubes; Table S2: Haematology parameters; Table S3: Physical measurements; Table S4: Immune parameters; Table S5: Phenotyping; Table S6: Serum cytokines; Table S7: Nucleotides.
