# Supplementary material for: Dietary Supplementation with Nucleotides, Short-Chain Fructooligosaccharides, Xylooligosaccharides, Beta-Carotene and Vitamin E Influences Immune Function in Kittens
Source: Animals (Basel). 2023 Dec 2;13(23):3734. doi: 10.3390/ani13233734 (PMC10705356; doi:10.3390/ani13233734)
Supplement: Supplementary file 1 [file animals-13-03734-s001.zip › animals-2701501 supplementary.pdf]

Supplementary Material

Dietary Supplementation with Nucleotides, Short-Chain Fructooligosaccharides, Xylooligosaccharides, Beta-Carotene and Vitamin E Influences Immune Function in Kittens

Supplementary Table S1: Sample collection volumes and blood tubes

Details of total blood collection volumes, measures and blood tubes per timepoint.

Week	Total blood at timepoint (ml)	Measures	Volume required for measure (ml)	Blood tube
10	3	Serum titres, biochemistry and immunoglobulin	2.2	2 x 1.1ml gel-activated serum clot
		Haematology	0.2	200µl K3-EDTA Microvette
		Nucleotides	0.6	500µl K3-EDTA Microvette and 200µl K3-EDTA Microvette
12	4	Serum titres, biochemistry, immunoglobulin and cytokines	2.7	3ml gel-activated serum clot
		Proliferation and phagocytosis	1.2	1.3ml Lithium Heparin tube
14 and 23	3	Serum titres, biochemistry, immunoglobulin and cytokines	2.7	3ml gel-activated serum clot tube
		Haematology	0.2	200µl K3-EDTA Microvette
18 and 27	4.7	Serum titres, biochemistry and immunoglobulin	2.2	2 x 1.1ml gel-activated serum clot
		Haematology	0.2	200µl K3-EDTA Microvette tube
		Nucleotides	0.6	500µl K3-EDTA Microvette and 200µl K3-EDTA Microvette
		Phenotyping	0.5	500µl K3-EDTA Microvette
		Proliferation and phagocytosis	1.2	1.3ml Lithium Heparin

Blood tubes
1.1ml gel-activated serum clot tubes (Sarstedt 41.1378.005)
200µl K3-EDTA Microvette tube (Sarstedt 20.1288)
500µl K3-EDTA Microvette 500 (Sarstedt 20.1341)
3ml gel-activated serum clot tube (Greiner Bio One, 454095)
1.3ml Lithium Heparin tube (Sarstedt, 41.1393.005)

Supplementary Table S2: Haematology parameters

Haematology values given as means and 95% confidence intervals (CI) for control and test kitten diet group. P values represents comparison between diet group at the sample collection timepoint, values in bold are statistically significant ($p < 0.05$).

Parameter	Weeks	Control			Test			*p*
		n	Mean	95% CI	n	Mean	95% CI	
Red blood cells (10^12/L)	10	15	7.649	7.200, 8.098	8	7.370	6.813, 7.926	0.430
	14	12	7.382	6.909, 7.856	14	7.866	7.366, 8.367	0.159
	18	13	7.400	6.939, 7.862	11	7.614	7.088, 8.141	0.534
	23	11	8.135	7.654, 8.615	15	8.917*	8.422, 9.413	0.028
	27	19	8.528*	8.107, 8.950	15	8.548*	8.054, 9.042	0.950
Haemoglobin (g/dL)	10	15	10.59	9.90, 11.29	8	11.09	10.24, 11.93	0.361
	14	12	10.11	9.39, 10.83	14	11.33	10.55, 12.12	0.027
	18	13	10.36	9.65, 11.07	11	11.36	10.54, 12.17	0.068
	23	11	11.51*	10.78, 12.24	15	12.31*	11.53, 13.10	0.130
	27	19	11.96*	11.30, 12.62	15	12.57*	11.79, 13.35	0.223
Hematocrit (l/L)	10	15	34.92	32.47, 37.38	8	36.77	33.79, 39.74	0.328
	14	12	33.40	30.87, 35.93	14	37.40	34.59, 40.21	0.039
	18	13	33.68	31.18, 36.17	11	36.65	33.77, 39.54	0.118
	23	11	36.57	34.02, 39.11	15	39.58*	36.78, 42.38	0.111
	27	19	38.18*	35.81, 40.56	15	40.49*	37.69, 43.28	0.199
Mean corpuscular volume (fL)	10	15	45.82	43.18, 48.45	8	49.83	46.68, 52.98	0.054
	14	12	45.33	42.66, 48.01	14	47.66	44.59, 50.72	0.238
	18	13	45.57	42.91, 48.22	11	48.01	44.91, 51.11	0.220
	23	11	45.15	42.46, 47.83	15	44.60*	41.54, 47.65	0.776
	27	19	44.90	42.31, 47.49	15	47.25*	44.19, 50.30	0.225
Mean corpuscular haemoglobin (pg)	10	15	13.91	13.17, 14.65	8	15.03	14.14, 15.92	0.057
	14	12	13.73	12.97, 14.48	14	14.46	13.60, 15.31	0.192
	18	13	14.01	13.26, 14.76	11	14.91	14.04, 15.78	0.114
	23	11	14.22	13.45, 14.98	15	13.91*	13.07, 14.76	0.579
	27	19	14.06	13.34, 14.78	15	14.68	13.83, 15.53	0.253
Mean corpuscular haemoglobin concentration (g/dL)	10	15	30.34	29.84, 30.83	8	30.22	29.61, 30.82	0.750
	14	12	30.27	29.76, 30.79	14	30.34	29.78, 30.90	0.853
	18	13	30.71	30.20, 31.22	11	31.09*	30.51, 31.67	0.313
	23	11	31.52*	31.00, 32.05	15	31.20*	30.64, 31.76	0.384
	27	19	31.35*	30.87, 31.82	15	31.10*	30.54, 31.65	0.480
Red blood cell distribution width (%)	10	15	24.02	22.91, 25.14	8	22.32	20.94, 23.71	0.060
	14	12	19.15*	17.97, 20.33	14	18.62*	17.38, 19.86	0.526
	18	13	16.98*	15.83, 18.13	11	16.79*	15.48, 18.10	0.822
	23	11	17.08*	15.88, 18.28	15	18.38*	17.15, 19.61	0.130
	27	19	16.64*	15.59, 17.68	15	17.11*	15.89, 18.33	0.545
White blood cells (10^9/L)	10	15	19.89	16.20, 23.58	8	12.97	8.50, 17.44	0.022
	14	12	15.16*	11.36, 18.97	14	10.68	6.45, 14.90	0.114
	18	13	13.21*	9.46, 16.96	11	10.12	5.78, 14.46	0.271
	23	11	13.98*	10.15, 17.81	15	11.33	7.12, 15.53	0.337
	27	19	14.35*	10.79, 17.92	15	10.25	6.05, 14.45	0.132
Lymphocytes (10^9/L)	10	15	10.050	8.107, 11.99	8	5.706	3.357, 8.055	0.008
	14	12	7.069*	5.072, 9.067	14	4.846	2.616, 7.076	0.134
	18	13	6.975*	5.004, 8.947	11	4.742	2.458, 7.025	0.136
	23	11	6.439*	4.427, 8.451	15	4.447	2.227, 6.668	0.177

	27	19	7.300*	5.418, 9.182		15	5.244	3.024, 7.465		0.151
Monocytes (10^9/L)	10	15	0.6779	0.5076, 0.8483		8	0.3164	0.1114, 0.5215		0.011
	14	12	0.4567*	0.2824, 0.6309		14	0.2927	0.0962, 0.4893		0.202
	18	13	0.4552*	0.2829, 0.6276		11	0.2709	0.0705, 0.4713		0.158
	23	11	0.4234*	0.2481, 0.5987		15	0.2674	0.0716, 0.4633		0.224
	27	19	0.4492*	0.2832, 0.6151		15	0.2874	0.0916, 0.4833		0.196
Total granulocytes (10^9/L)	10	15	9.191	7.291, 11.090		8	6.955	4.627, 9.283		0.137
	14	12	7.642	5.661, 9.623		14	5.528	3.383, 7.672		0.146
	18	13	5.775*	3.833, 7.717		11	5.126	2.897, 7.354		0.652
	23	11	7.141	5.138, 9.144		15	6.605	4.476, 8.735		0.705
	27	19	6.615*	4.807, 8.424		15	4.772	2.642, 6.901		0.181
Platelet count (10^9/L)	10	15	585.7	423.1, 810.9		8	542.7	360.3, 817.5		0.769
	14	12	569.1	402.0, 805.7		14	510.1	356.6, 729.7		0.656
	18	13	282.4*	201.6, 395.5		11	343.3	234.3, 503.0		0.440
	23	11	503.7	353.7, 717.4		15	438.4	307.9, 624.4		0.574
	27	19	331.0*	244.9, 447.4		15	357.8	251.6, 508.8		0.731
Mean platelet volume (fL)	10	15	10.27	9.59, 10.95		8	9.51	8.69, 10.33		0.153
	14	12	9.82	9.12, 10.52		14	9.28	8.50, 10.06		0.291
	18	13	9.47*	8.78, 10.17		11	8.98	8.17, 9.78		0.334
	23	11	9.40*	8.69, 10.10		15	9.06	8.28, 9.84		0.507
	27	19	9.60*	8.94, 10.25		15	9.37	8.59, 10.15		0.639

* A significant difference from week 10 within diet group ($p < 0.05$)

Supplementary Table S3: Physical measurements

Physical measurement values given as means and 95% confidence intervals (CI) for control and test kitten diet group. P values represents comparison between diet group at the sample collection timepoint, values in bold are statistically significant (p < 0.05).

**Parameter**	**Weeks**	**Control**				**Test**				***p***
		**n**	**Mean**	**95% CI**		**n**	**Mean**	**95% CI**		
Height at withers (cm)	27	20	26.8	25.8, 27.8		20	27.6	26.5, 28.7		0.263
Thoracic girth (cm)	27	20	29.3	28.3, 30.3		20	30.9	30.2, 31.7		0.013
Rump width (cm)	27	20	7.1	6.3, 8.0		20	6.9	5.9, 8.0		0.789

Supplementary Table S4: Immune parameters

Immune parameter values given as means and 95% confidence intervals (CI) for control and test kitten diet group. P values represents comparison between diet group at the sample collection timepoint, values in bold are statistically significant ($p < 0.05$).

Parameter	Weeks	Control			Test			*p*
		n	Mean	95% CI	n	Mean	95% CI	
Phagocytosis (%)	12	11	60.3	47.1, 73.5	12	55.6	41.4, 69.7	0.602
	18	19	53.4	44.6, 62.1	14	59.9	48.8, 70.9	0.330
	27	20	49.3	39.9, 58.7	14	61.8	50.4, 73.3	0.089
Phenotyping: CD5+ cells (%)	18	7	16.8	6.4, 27.1	6	25.3	12.7, 37.9	0.233
	27	8	19.8	9.7, 29.9	7	29.0	17.6, 40.4	0.180
Phenotyping: CD5+CD4+ cells (%)	18	7	10.6	3.3, 17.9	7	18.7	9.9, 27.6	0.130
	27	8	12.7	5.6, 19.9	8	17.2	9.1, 25.3	0.327
Phenotyping: CD21+ cells (%)	18	7	18.2	12.1, 24.3	6	22.4	15.3, 29.5	0.293
	27	8	16.0	10.3, 21.7	7	24.8	18.2, 31.4	0.054
Phenotyping: CD5+CD8+ cells (%)	18	7	3.3	1.7, 4.9	6	4.8	2.6, 7.0	0.209
	27	8	8.1*	3.2, 13.1	7	8.7*	3.7, 13.6	0.843
Phenotyping CD5+CD4+: CD5+CD8+	18	7	3.1	2.3, 3.9	6	3.6	2.6, 4.6	0.360
	27	8	2.0*	1.1, 2.9	7	2.1*	1.1, 3.0	0.840
Proliferation: SEB EC50 (cell count/ml)	12	12	200000	57966, 342063	12	24797	-120990, 170585	0.089
	18	14	191488	62913, 320062	9	288800*	138885, 438715	0.320
	27	9	226497	107841, 345153	5	157873	14775, 300970	0.454
Proliferation: SEB EC80 (cell count/ml)	12	12	412700	223232, 602168	12	146152	-46145, 338449	0.052
	18	14	401116	230658, 571575	9	556351*	358448, 754255	0.233
	27	9	416167	259798, 572537	5	437285*	248631, 625939	0.861
Proliferation: Con A EC50 (cell count/ml)	12	12	227170	90218, 364121	12	133482	-9993, 276958	0.340
	18	14	257185	128212, 386158	9	170427	23168, 317685	0.368
	27	9	229067	104743, 353392	5	175202	34188, 316215	0.559
Proliferation: Con A EC80 (cell count/ml)	12	12	231692	61560, 401824	12	177846	-4502, 360194	0.659
	18	14	224400	68437, 380364	9	203404	16944, 389864	0.859
	27	9	264307	114750, 413864	5	253435	73738, 433131	0.923
Proliferation: PMA/ION EC50 (cell count/ml)	12	12	146776	-42815, 336367	12	-1919	-200086, 196249	0.276
	18	14	117180	-56251, 290611	9	64706	-138686, 268097	0.689
	27	9	279894	118199, 441588	5	117349	-77418, 312116	0.197
Proliferation: PMA/ION EC80 (cell count/ml)	12	12	326615	127910, 525320	12	42890	-151887, 237666	0.046
	18	14	484610	308877, 660344	9	695310*	494672, 895949	0.117
	27	9	396862	238371, 555353	5	413366*	222243, 604489	0.893

SEB, Staphylococcal Enterotoxin B; Con A, Concanavalin A; PMA/ION, Phorbol 12-myristate 13-acetate/Ionomycin

* A significant difference from first timepoint within diet group ($p < 0.05$)

Supplementary Table S5: Phenotyping

Immune parameter values given as medians and interquartile range (IQR) for control and test kitten diet group. P values represents comparison between diet group at the sample collection timepoint, values in bold are statistically significant ($p < 0.05$).

Parameter		Control				Test				p
	Weeks	n	Median	IQR		n	Median	IQR		
Phenotyping:	18	7	33.6	31.4, 36.7		6	41.7	36.6, 42.9		0.474
CD18+ cells (%)	27	8	39.3	28.9, 46.3		7	44.3	26.6, 72.5		0.743

A significant difference from first timepoint within diet group ($p < 0.05$)

Supplementary Table S6: Serum cytokines

Serum cytokine values given as means and 95% confidence intervals (CI) for control and test diet kitten group. P values represents comparison between diet group at the sample collection timepoint (*p* < 0.05 is statistically significant).

Parameter		Control				Test				*p*
	Week	n	Mean	95% CI		n	Mean	95% CI		
Flt-3L (pg/ml)	12	9	33.07	22.29, 43.85		11	45.28	34.51, 56.06		0.103
	14	6	32.95	21.59, 44.30		12	24.04*	13.33, 34.75		0.231
	23	9	34.73	23.98, 45.47		7	21.13*	10.12, 32.15		0.077
GM CSF (pg/ml)	12	2	16.96	-4.61, 38.53		6	29.99	11.40, 48.59		0.259
	14	2	32.90*	11.36, 54.45		1	28.59	9.95, 47.23		0.700
	23	1	15.67*	-5.59, 36.93		2	56.12*	38.98, 73.26		0.010
IFN-γ (pg/ml)	12	10	207.4	79.6, 335.2		8	165.9	16.7, 315.0		0.663
	14	7	266.4	122.6, 410.1		11	197.9	61.0, 334.8		0.478
	23	10	191.7	59.9, 323.4		10	93.2	-45.2, 231.6		0.291
IL-1β (pg/ml)	12	7	28.18	13.06, 43.29		9	51.92	36.17, 67.66		0.036
	14	4	33.46	15.87, 51.06		12	14.70*	-0.34, 29.73		0.100
	23	7	24.86	9.46, 40.25		8	15.75*	0.27, 31.22		0.369
IL-2 (pg/ml)	12	6	30.70	15.16, 62.15		10	67.21	30.91, 146.10		0.122
	14	5	41.47	20.06, 85.75		8	29.94*	13.62, 65.83		0.506
	23	3	32.72	15.96, 67.09		7	23.10*	10.54, 50.65		0.473
PDGF-BB (pg/ml)	12	12	1850	982, 2718		15	2267	1255, 3279		0.506
	14	10	1944	1065, 2822		13	1740*	729, 2752		0.746
	23	11	1824	956, 2691		15	1703*	693, 2712		0.845
IL-12 (pg/ml)	12	12	152.3	113.3, 191.4		15	202.9	158.8, 247.1		0.087
	14	12	145.9	105.5, 186.2		15	154.2*	110.1, 198.2		0.771
	23	15	153.2	114.4, 191.9		15	138.2*	94.4, 182.1		0.592
IL-13 (pg/ml)	12	9	22.46	17.32, 29.11		11	28.60	21.39, 38.24		0.205
	14	9	23.49	18.05, 30.58		12	17.76*	13.35, 23.61		0.145
	23	11	19.36	15.00, 24.99		13	20.41*	15.26, 27.30		0.774
IL-4 (pg/ml)	12	10	281.6	121.2, 654.2		12	173.4	66.1, 455.0		0.428
	14	12	207.2	87.7, 489.7		12	182.5	69.8, 476.8		0.834
	23	11	173.7	74.8, 403.4		13	165.8	63.3, 434.1		0.938
IL-6 (pg/ml)	12	12	108.8	51.9, 228.0		10	84.2	35.8, 197.9		0.635
	14	12	86.2	39.9, 186.2		15	85.1	36.8, 196.6		0.980
	23	10	75.5	35.6, 159.8		10	81.8	35.1, 190.3		0.882
IL-8 (pg/ml)	12	7	36.96	19.99, 53.93		7	54.09	35.13, 73.04		0.160
	14	6	36.33	19.09, 53.56		6	16.77*	-2.44, 35.98		0.122
	23	3	33.40	14.45, 52.35		3	25.25*	2.61, 47.88		0.566
KC (pg/ml)	12	1	20.85	2.87, 38.83		1	15.63	-11.75, 43.01		0.746
	14	4	17.87	4.57, 31.18		3	19.69	8.13, 31.25		0.813
	23	6	32.54	21.77, 43.32		4	32.13	16.29, 47.98		0.948
SDF 1 (pg/ml)	12	12	1079	767, 1391		14	1131	779, 1483		0.816
	14	12	1222	900, 1544		15	1093	742, 1444		0.571
	23	14	1159	850, 1469		15	1108	758, 1457		0.815
RANTES (pg/ml)	12	7	24.58	16.87, 35.83		9	30.10	19.96, 45.40		0.442
	14	8	23.59	15.96, 34.86		8	15.29*	10.01, 23.36		0.128
	23	9	21.38	14.51, 31.52		6	18.93*	12.46, 28.75		0.651
SCF (pg/ml)	12	12	60.13	40.21, 89.91		12	42.20	26.66, 66.80		0.236

	14	11	51.23	33.69, 77.92		13	42.11	26.58, 66.70		0.514
	23	14	47.26	31.75, 70.34		13	35.70	22.71, 56.13		0.335
TNF_a (pg/ml)	12	12	138.4	18.1, 258.6		12	220.3	57.7, 383.0		0.306
	14	14	192.7	70.8, 314.7		9	111.7	-96.6, 320.0		0.408
	23	9	54.7	-69.6, 178.9		5	61.6*	-93.3, 216.5		0.924
IL-18 (pg/ml)	12	12	436.7	159.4, 713.9		11	720.7	408.3, 1033.0		0.167
	14	9	452.1	165.5, 738.7		13	85.2*	-226.2, 396.5		0.084
	23	11	281.1	6.4, 555.9		12	133.5*	-176.7, 443.8		0.456

Flt-3L, FMS-related tyrosine kinase 3 ligand; GM CSF, Granulocyte-macrophage colony-stimulating factor; IFN-γ, Interferon gamma; IL-1β, Interleukin-1 beta; IL-2, Interleukin-2; PDGF-BB, Platelet-derived growth factor-BB; IL-12, Interleukin-12; IL-13, Interleukin-13; IL-4, Interleukin-4; IL-6, Interleukin-6; IL-8, Interleukin-8; KC, Keratinocyte chemoattractant; SDF 1, Stromal cell-derived factor 1; RANTES, Regulated on activation, normal T cell expressed and secreted; SCF, Stem cell factor; TNF_a, Tumour Necrosis Factor alpha; IL-18, Interleukin-18

* A significant difference from baseline within diet group ($p < 0.05$)

Supplementary Table S7: Nucleotides

Blood nucleotides values given as means and 95% confidence intervals (CI) for control and test diet kitten group. P values represents comparison between diet group at the sample collection timepoint ($p < 0.05$ is statistically significant).

Parameter		Control				Test				p
	Week	n	Mean	95% CI		n	Mean	95% CI		
GMP (nmol/ml)	10	14	2.21	0.71, 3.71		11	2.18	0.54, 3.81		0.967
	18	16	2.72	1.27, 4.17		11	2.48	0.84, 4.11		0.772
	27	12	0.98*	-0.55, 2.51		14	1.67	0.11, 3.24		0.408
IMP (nmol/ml)	10	14	0.318	0.155, 0.482		11	0.239	0.055, 0.423		0.470
	18	16	0.244	0.091, 0.398		11	0.235	0.052, 0.419		0.933
	27	12	0.282	0.108, 0.456		14	0.301	0.133, 0.469		0.859
AMP (nmol/ml)	10	14	10.40	6.32, 14.50		11	7.40	3.00, 11.80		0.149
	18	16	7.99	3.99, 12.00		11	7.14	2.74, 11.50		0.681
	27	12	8.58	4.42, 12.70		14	6.06	1.80, 10.30		0.225
CDP (nmol/ml)	10	14	1.50	0.94, 2.06		11	1.43	0.83, 2.02		0.794
	18	16	1.28	0.74, 1.83		11	1.13	0.53, 1.72		0.556
	27	12	0.35*	-0.21, 0.92		14	0.62*	0.03, 1.20		0.330
UDP (nmol/ml)	10	14	1.08	0.74, 1.43		11	0.63	0.25, 1.02		0.049
	18	16	0.53*	0.20, 0.85		11	0.50	0.11, 0.89		0.901
	27	12	1.25	0.89, 1.61		14	0.83	0.47, 1.19		0.058
GDP (nmol/ml)	10	14	4.01	3.26, 4.76		11	3.95	3.00, 4.90		0.895
	18	16	2.99*	2.26, 3.72		11	3.66	2.71, 4.61		0.163
	27	12	3.28*	2.52, 4.05		14	3.34	2.44, 4.24		0.903
CTP (nmol/ml)	10	4	0.969	0.458, 1.480		4	0.995	0.458, 1.530		0.936
	18	5	0.783	0.328, 1.240		4	0.164*	-0.373, 0.701		0.079
	27	8	0.350*	-0.023, 0.723		7	0.266*	-0.184, 0.716		0.745
ADP (nmol/ml)	10	14	36.7	30.0, 43.5		11	32.3	24.8, 39.8		0.298
	18	16	29.8*	23.4, 36.2		11	27.4	19.9, 34.9		0.556
	27	12	40.8	33.8, 47.8		14	35.0	28.0, 42.0		0.166
UTP (nmol/ml)	10	14	7.63	4.98, 10.30		11	7.67	4.86, 10.50		0.970
	18	16	7.06	4.71, 9.41		11	5.00*	2.50, 7.50		0.040
	27	12	4.79*	2.49, 7.09		14	3.85*	1.49, 6.22		0.285
GTP (nmol/ml)	10	14	8.66	6.93, 10.40		11	8.81	6.91, 10.70		0.886
	18	16	6.84*	5.29, 8.39		11	8.15	6.37, 9.93		0.176
	27	12	5.56*	4.03, 7.08		14	6.39*	4.83, 7.95		0.336
ATP (nmol/ml)	10	14	149	128, 170		11	135	112, 159		0.292
	18	16	157	137, 178		11	148	125, 172		0.472
	27	12	155	133, 177		14	143	120, 165		0.328
UMP,UDP and UTP (nmol/ml)	10	14	10.30	7.55, 13.00		11	8.51	5.62, 11.40		0.148
	18	16	8.65	6.14, 11.20		11	6.06*	3.40, 8.71		0.011
	27	12	7.32*	4.84, 9.80		14	5.37*	2.83, 7.91		0.034
GMP, GDP and GTP (nmol/ml)	10	14	14.8	12.3, 17.4		11	15.1	12.3, 17.9		0.874
	18	16	12.5	10.1, 14.9		11	14.4	11.6, 17.2		0.209
	27	12	9.8*	7.2, 12.4		14	11.5*	8.89, 14.2		0.252
AMP, ADP and ATP (nmol/ml)	10	14	148	127, 172		11	133	112, 157		0.239
	18	16	154	133, 178		11	145	123, 172		0.537
	27	12	154	132, 181		14	140	120, 164		0.291
	10	13	2.98	1.68, 4.28		8	0.79	-0.59, 2.16		0.000

C nucleosides (nmol/ml)	18	12	3.20	1.90, 4.50		11	1.29	-0.04, 2.62		0.002
	27	12	3.63	2.34, 4.91		14	1.29	-0.01, 2.58		0.000
U nucleosides (nmol/ml)	10	13	37.7	22.6, 52.8		8	17.4	0.7, 34.1		0.003
	18	12	33.3	18.1, 48.4		11	19.6	3.4, 35.8		0.039
	27	12	36.0	21.0, 51.1		14	19.3	3.5, 35.1		0.011
G nucleosides (nmol/ml)	10	13	17.3	14.2, 20.5		8	16.7	13.1, 20.3		0.749
	18	12	16.5	13.4, 19.7		11	14.8	11.5, 18.1		0.342
	27	12	13.9*	10.7, 17.0		14	12.3*	9.2, 15.4		0.376
A nucleosides (nmol/ml)	10	13	184	162, 206		8	171	146, 196		0.346
	18	12	194	172, 217		11	170	146, 193		0.064
	27	12	186	163, 208		14	170	148, 192		0.230
Total nucleosides (nmol/ml)	10	13	242	207, 277		8	207	169, 246		0.076
	18	12	248	213, 283		11	207	171, 244		0.036
	27	12	239	204, 273		14	205	170, 240		0.074

GMP, Guanosine monophosphate; IMP, Inosine monophosphate; AMP, Adenosine monophosphate; CDP, Cytidine diphosphate; UDP, Uridine diphosphate; GDP, Guanosine diphosphate; CTP, Cytidine triphosphate; ADP, Adenosine diphosphate; UTP, Uridine triphosphate; GTP, Guanosine triphosphate; ATP, Adenosine triphosphate; UMP, Uridine monophosphate; C Nucleosides, Cytidine nucleosides; U Nucleosides, Uridine nucleosides; G Nucleosides, Guanosine nucleosides; A Nucleosides, Adenosine nucleosides

* A significant difference from week 10 within diet group ($p < 0.05$)
